# Composition and Functionality of Lipid Emulsions in Parenteral Nutrition: Examining Evidence in Clinical Applications

**DOI:** 10.3389/fphar.2020.00506

**Published:** 2020-04-29

**Authors:** Birinder Kaur Sadu Singh, Sreelakshmi Sankara Narayanan, Ban Hock Khor, Sharmela Sahathevan, Abdul Halim Abdul Gafor, Enrico Fiaccadori, Kalyana Sundram, Tilakavati Karupaiah

**Affiliations:** ^1^ Nutrition Programme, Faculty of Health Sciences, National University of Malaysia, Kuala Lumpur, Malaysia; ^2^ Faculty of Health & Medical Science, School of BioSciences, Taylor’s University Lakeside Campus, Selangor, Malaysia; ^3^ Dietetics Programme, Faculty of Health Sciences, National University of Malaysia, Kuala Lumpur, Malaysia; ^4^ Medical Department, Faculty of Medicine, Universiti Kebangsaan Malaysia Medical Centre, Kuala Lumpur, Malaysia; ^5^ Acute and Chronic Renal Failure Unit, Department of Clinical and Experimental Medicine, University of Parma, Parma, Italy; ^6^ Malaysian Palm Oil Council (MPOC), Petaling Jaya, Malaysia

**Keywords:** fatty acids, lipid emulsions, triglycerides, triacylglycerols, parenteral nutrition

## Abstract

Lipid emulsions (LEs), an integral component in parenteral nutrition (PN) feeding, have shifted from the primary aim of delivering non-protein calories and essential fatty acids to defined therapeutic outcomes such as reducing inflammation, and improving metabolic and clinical outcomes. Use of LEs in PN for surgical and critically ill patients is particularly well established, and there is enough literature assigning therapeutic and adverse effects to specific LEs. This narrative review contrarily puts into perspective the fatty acid compositional (FAC) nature of LE formulations, and discusses clinical applications and outcomes according to the biological function and structural functionality of fatty acids and co-factors such as phytosterols, α-tocopherol, emulsifiers and vitamin K. In addition to soybean oil-based LEs, this review covers clinical studies using the alternate LEs that incorporates physical mixtures combining medium- and long-chain triglycerides or structured triglycerides or the unusual olive oil or fish oil. The Jaded score was applied to assess the quality of these studies, and we report outcomes categorized as per immuno-inflammatory, nutritional, clinical, and cellular level FAC changes. It appears that the FAC nature of LEs is the primary determinant of desired clinical outcomes, and we conclude that one type of LE alone cannot be uniformly applied to patient care.

## Introduction

Fundamental knowledge in lipid science is well elucidated in terms of the fat molecule’s contribution to total caloric needs, metabolic pathways of energy production, utilization, and storage in the human system. Further, the differentiation of fats based on chemical structure has led to an understanding on metabolic risks associated with atherogenecity in the non-critical status (Karupaiah et al., 2005). On the other hand, benefits of lipid emulsions (LEs) in parenteral therapy has been evolving since the 1960s. I interestingly, although LEs are been administered intravenously in critical care and long-term nutrition support, there is little knowledge regarding the compositional nature and metabolic outcomes related to the nature of lipids in these formulations. Lipids were a late macronutrient addition to parenteral nutrition (PN) formulation in the 1960s, with experimentation leading to formulation of a broad range of intravenous LE products from variable fat sources (Wretlind, 1981).

The first commercially available LE in 1961 was based exclusively on soybean oil, contributing ~ 50% from *n*-6 linoleic acid (LA) in its total fatty acid (FA) profile. But side effects related to the high LA content added to a higher oxidative stress burden in critically ill patients (Calder et al., 2010) leading to a decision to reduce the LA content in LEs, and opening the way for alternative LEs in parenteral applications (Waitzberg et al., 2006). This development offers new challenges evidenced from emerging clinical studies with aspects relating to inflammation (Calder, 2012; Miles and Calder, 2015), immunomodulation (Waitzberg et al., 2002), and clinical outcomes (Calder, 2013) been reviewed in specific patient populations. However, a collective examination of the functionality of LEs relating to the structure and composition of lipids and their properties to metabolic outcomes has not been related to parenteral therapy in literature. In contrast, core knowledge on metabolic effects of stereospecific positioning of FAs in triglyceride molecules in native and structured fats has been reviewed in the context of atherogenic and metabolic risk (Karupaiah and Sundram, 2007) in healthy humans.

Therefore, this review highlights the composition of FAs in LEs, other components in LEs, biological function of FAs, the metabolic properties of FAs in different LEs, and clinical evidence associated with the use of these LEs in surgery, critically ill, and long-term PN patients.

### Fatty Acids

The functionality of the triacylglycerol (TAG) molecules is determined by the type of FA esterified to the glycerol backbone and the stereospecificity of the linkage (Karupaiah and Sundram, 2007). Although FAs share the common structural formula of CH3(CH2)_X_COOH, the chain length of carbon atoms linked to the methyl and carboxyl groups varies (Gunstone et al., 2007), with 4 carbon atoms or less termed as short-chain FAs, 6–14 carbon atoms as medium chain, and above 14 carbon atoms classified as long-chain FAs. Again FAs can be reclassified according to the presence or absence of saturation between carbon bonds. The saturated FAs (SFAs) belonging to medium-chain triglycerides (MCTs) feature in some LE formulations. Longer carbon chains with the 22-carbon docosahexaenoic acid (DHA) and the 20-carbon eicosapentaenoic acid (EPA) are also known in LE formulations. Saturation and unsaturation between carbon atoms will determine the functionality and usability of the FAs (Calder, 2004; Kim et al., 2010; Calder, 2012; Calder, 2013; Miles and Calder, 2015). Thus, in discussing FAs, their classification according to carbon chain length, degree of saturation or unsaturation, and the location of double bonds need to be appreciated in the context of LEs.

The melting point of saturated oils used in LEs increase with increasing FAs chain length (Gunstone et al., 2007). Physically MCTs are liquid at room temperature (Ulrich et al., 1996) and have a higher melting point compared to unsaturated FAs. For this reason MCTs are blended with other long-chain FAs such as oleic acid, LA, α-linolenic acid, DHA, and EPA to reduce the melting point to facilitate administration via the intravenous route (**Table 1**). Contrarily, the presence of double bonds in the carbon chain length lowers the melting point which increases the liquidity of lipid at room temperature (Gunstone et al., 2007) (**Table 1**). Therefore the unsaturated MUFAs and polyunsaturated fatty acids (PUFAs) benefit lower melting points enabling the liquid state of LEs at room temperature compared to the more saturated MCTs.

**Table 1:** Melting point of commonly used fatty acids in lipid emulsions (Gunstone et al., 2007).

Chain length	Fatty acids	Melting point
C12:0	Lauric acid	44.8°C
C18:1	Oleic acid	13.4°C
C18:2	Linoleic acid	−5.0°C
C18:3	α-Linolenic acid	−11.0°C

### Other Components in LEs

Other microcomponents in LEs such as phytosterols, α-tocopherol, emulsifiers, and vitamin K further confer functional characteristics. In practice, Vanek *et al*. (2012) has noted information on these additives may not be declared on the LE product label depending on country availability and local regulation.

#### Phytosterols

Phytosterols are plant-based and include sitosterol, campesterol, and stigmasterol, sharing a similar structure with cholesterol. In normal metabolism, less than 5% of sterols are absorbed with the rest entering the colon (Ling and Jones, 1995). However in children receiving PN infusion, Clayton *et al.* (1993) reported occurrence of cholestatic liver disease, likely attributed to the 100% uptake of phytosterols into systemic circulation. In this situation, liver metabolism favors the conversion of sterols to bile salts, which been less soluble than cholesterol, is likely to be precipitated. The accumulation of sterol precipitates within liver cells is the hallmark of cholestasis (Clayton et al., 1993). Sterol precipitation inhibits rate-limiting cholesterol 7α-hydroxylase in the liver affecting bile acid secretion (Boberg et al., 1989). Pianese et al. (2008) reported that accumulation of phytosterols in newborns’ plasma and red blood cell membrane may induce PN-related cholestasis. 

Indeed intravenous infusion of phytosterols in animal models, indicate its accumulation in the blood, liver, and bile, causing cholestasis (Iyer et al., 1998). Cholestasis emerged as an issue with long-term use of LEs when incorporating olive oil in infants (Savini et al., 2013) or soybean oil in children (Clayton et al., 1993).

#### α-Tocopherol

The main form of vitamin E used in LEs is tocopherol occurring as α, β, γ-, or ơ-isoforms (Gurr, 1992). Addition of α-tocopherol into LEs is to prevent peroxidation in susceptible PUFA-rich lipids (Biesalski, 2009) because of unsaturated double-bonds (Pironi et al., 2003).

Wanten et al. (2002) evaluated tocopherol isoform applications, and concluded that long-chain triglycerides (LCTs) in LEs formulated from soybean and safflower oils are predominantly γ-tocopherol, which provide little protection against lipid peroxidation compared to fish oil LE (FOLE) with the highest concentration of α-tocopherol (Wanten et al., 2002). Therefore it is reasonable to increase the α-tocopherol concentration to protect soybean oil LEs against lipid peroxidation. In order to maintain LE stability, addition of α-tocopherol requires a separate administration (Steger and Muhlebach, 1998). Thus, the tocopherol content of LEs is a critical factor in patient administration.

In animal models, some vitamin E isoforms show benefit in prevention of hepatic injury. Ng et al. (2016) reported the use of α-tocopherol in pre-term piglets causing reduction in serum markers of hepatic injury and cholestasis. However, Raphael and Duggan (2012) observed that tocopherol alone was not effective in treatment and prevention of intestinal failure associated liver disease in infants. Diamond et al. (2009) proposed several mechanisms for the reduction of intestinal failure-associated liver disease in children using fish oil-based LE, reduced phytosterol load, increased α-tocopherol and *n*-3 PUFAs (DHA and EPA) content.

#### Emulsifiers

LEs consist of two immiscible liquid phases, oil in water. Phospholipids play the role of emulsifying agent to allow the dispersion of fat droplets in the aqueous phase (Driscoll, 2015). Main sources of phospholipid are soybean oil and egg yolk. Lecithin from egg yolk has non-polar (lipophilic) and polar (hydrophilic) properties, which allow the dispersion of the fat droplets in aqueous phase of the emulsion (Ferezou and Bach, 1999; Driscoll, 2015). Fat droplets are prevented from coalescing by an electrostatic barrier generated by the anionic charge of phospholipid polar ends dissociating toward the aqueous phase, enabling stability of LEs (Driscoll, 2015).

With excess emulsifier, liposomes are generated in vesicular form termed as phospholipid-rich particles, forming a bilayer in the aqueous phase of LE. The liposome content is greater in LEs with a lower percentage of oil due to the higher phospholipid:oil ratio (Ferezou et al., 1994; Ferezou and Bach, 1999). The greater liposomes generation potentiates inhibition of lipolysis of artificial chylomicron, and also contributes to abnormal lipoproteins such as Lipoprotein-X. Lipoprotein-X causes hypercholesterolemia, cholesterol accumulation, and increase in free cholesterol:esterified cholesterol plasma ratio in human studies (Ferezou and Bach, 1999). Therefore the extent of liposome metabolism disturbance in plasma depends on the oil concentration in the LE, infusion rate, and duration of infusion (Hultin et al., 1994; Driscoll, 2015). It is noted that liposomes with diameter of <80 nm have no purpose as an energy source (Ferezou and Bach, 1999).

#### Vitamin K

Phylloquinone or vitamin K1 is important for normal blood clotting, and a deficiency would prolong the clotting time (Gurr, 1992). The dietary recommendation for vitamin K is 1 μg/kg for adults (Shearer, 2009) but for patients on anticoagulant therapy, the content of vitamin K1 in LEs should be considered when prescribing coumarin-based anticoagulants (Raman et al., 2017). The vitamin K content per 100 g of lipids used in LE formulation varies (Shearer, 2009; Raman et al., 2017), as indicated for olive oil (1.1–5.5 μg), soybean oil (150–300 μg), and safflower oil (6–12 μg).

## Biological Function of LEs

### Absorption and Transport of LEs

FAs introduced intravenously bypass the intestinal lumen and directly enter blood circulation. This means bypassing digestion by lipases, solubilization by bile, uptake into intestinal enterocytes, or packaging into chylomicrons compatible for circulatory transport to target organs. Lipids infused intravenously are already packaged into artificial chylomicrons ready for transport to target organs (Carpentier and Dupont, 2000). LE chylomicrons mimic the structural similarity of natural chylomicrons produced by intestinal enterocytes (Carpentier and Dupont, 2000) excepting the spherical size, as LE chylomicrons vary between 200 and 500 nm depending on the type of oil and its concentration in the LE (Lutz et al., 1989; Ferezou and Bach, 1999). With LCTs mean particle diameter is larger than for MCTs, and droplets in 20% emulsions are larger than with 10% emulsions (Lutz et al., 1989). Ease of passage of LE chylomicrons through the smallest of capillaries is essential to prevent vascular occlusion and is enabled with particle size within 200–500 nm (Driscoll et al., 2001).

LE chylomicrons are hydrolyzed to yield FAs by lipoprotein lipase, and then transported to the liver to provide energy substrate similar to the fate of natural chylomicrons (Carpentier and Dupont, 2000). Differentially, LCTs are transported in blood as chylomicrons while MCTs from partial uptake become free FAs which bind to albumin. The composition of the LE particle will influence the rates of hydrolysis and uptake of FAs as determined by the FA carbon chain length and the relative position of the FA on the glycerol backbone (Karupaiah and Sundram, 2007). Therefore, the shorter carbon chain length of MCTs are hydrolyzed by lipases at a faster rate compared to LCTs (Deckelbaum et al., 1990).

### Metabolism of FAs in LEs

Transported long-chain FAs are activated at the outer mitochondrial membrane by adenosine tri-phosphate to form acyl-adenylates before catalysis by acyl-CoA synthase. The sulfhydryl group of CoA reacts with the acyl-adenylate to form acyl-CoA. Aided by carnitine, the activated long-chain FAs then get transported into the mitochondrial matrix. The acyl carnitine formed from the reaction of acyl-CoA with the hydroxyl group of carnitine diffuses into the inner mitochondrial membrane. The acyl group of acyl-CoA reacts with the hydroxyl group of carnitine to form acyl-carnitine which enables diffusion into the inner mitochondrial membrane. Once inside the mitochondrial membrane, the acyl group will be transferred back to acyl-CoA and carnitine is released. These transacylation reactions are catalyzed by fatty acyl-CoA:carnitine FA transferase (Berg et al., 2012).

Medium-chain FAs been saturated and having shorter carbon chains generate energy faster, as the route of its fat oxidation differs from the longer FAs. These FAs promote passive movement across the mitochondrial double membrane, independent from carnitine allowing for direct oxidation (Ulrich et al., 1996; Gunstone et al., 2007). Metabolically, single-source MCT infusion gives rise to ketone bodies, predominantly β-hydroxybutyrates, as incomplete oxidation of medium-chain FAs in the mitochondria via β-oxidation converts intermediate metabolites of acetyl-CoA to ketones (Ball, 1993; Ulrich et al., 1996).

As regards structured triglycerides (STG), symmetrical TAGs formed by medium-chain FAs esterified at the *sn*-1 and *sn*-3 positions of the glycerol backbone would facilitate faster hydrolysis by lipoprotein lipase (Hultin et al., 1994; Karupaiah and Sundram, 2007). This facilitates faster uptake of long-chain monoglycerides into the cell, and medium-chain FAs to be transported into mitochondria for oxidation (Hultin et al., 1994).

**Figure 1:**
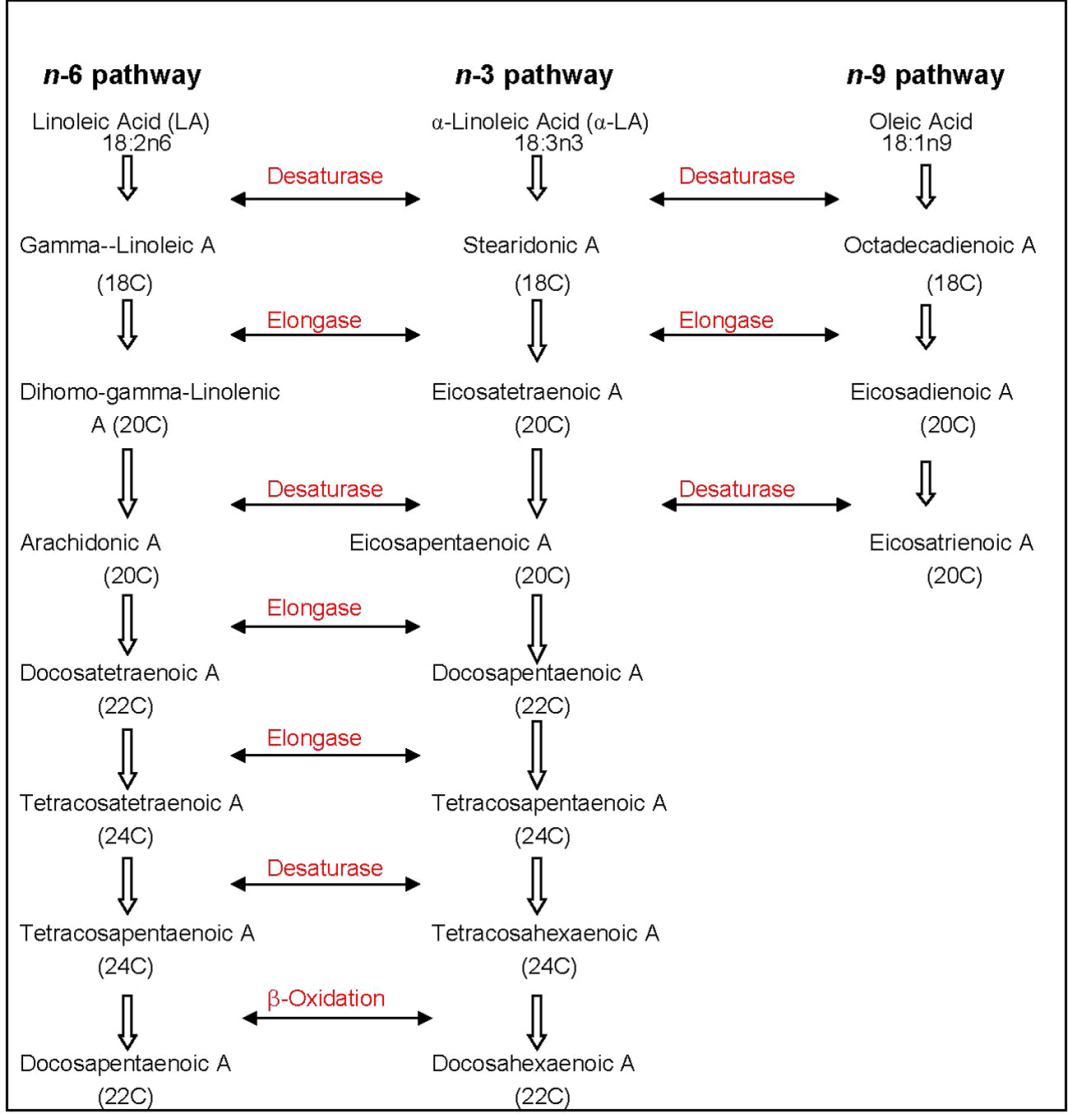
Metabolic pathway of *n*-3 and *n*-6 PUFAs with desaturase and elongase enzymes. The affinity for *n*-6 PUFAs by these enzymes are higher compared to *n*-9; A, acid; C, carbon; PUFA, polyunsaturated fatty acid. References (Gurr, 1992; Vanek et al., 2012).

The metabolic process of assimilating *n*-3 or *n*-6 PUFAs is competitive depending on their concentration and incorporating desaturation and chain elongation (Gunstone et al., 2007). The pathways of metabolism are shown in **Figure 1**. The preferential order of FA affinity for enzyme competition is in the order of α-linolenic > linoleic > oleic acid (Gurr, 1992). With essential FA deficiency, a third FA route utilizing oleic acid, becomes an option in the biological system (Vanek et al., 2012).

## Functionality of LEs

Unlike SFAs and MUFAs, humans are unable to synthesize PUFAs endogenously, particularly α-linolenic acid and LA, due to the lack of Δ-12 and Δ-15 desaturases which enable chain elongation (Gunstone et al., 2007; Wanten and Calder, 2007). Therefore supplementation of these FAs is vital in LEs. When α-linolenic acid and LA are supplemented, they undergo systemic chain elongation and desaturation driven by liver enzymes to produce a series of longer chain FAs which acquire additional double bonds (Wanten and Calder, 2007). Both these PUFAs maintain membrane structure and fluidity, tissue permeability and cell signaling, and are precursors of important lipid mediators (Calder and Grimble, 2002; Wanten and Calder, 2007). EPA and DHA generated from the chain elongation of α-linolenic acid biologically become structural components of cell membranes, especially in the brain and the retina.

Oleic acid originating from olive oil is the major form of MUFA in LEs and even available as free FAs. When LA is scarce and oleic acid is available in excess, oleic acid will competitively inhibit enzymes involved in arachidonic acid formation (Delgado et al., 2017). Evidence from animal and human studies suggest that PUFA and MUFA increase the activity of lipoprotein lipase which enhances TAG clearance during postprandial lipemia (Williams, 1997). In contrast, MCTs in LEs sourced from coconut oil carry shorter carbon chain FAs that are saturated, allowing ready oxidization in mitochondria and utilization as fuel for energy (Ulrich et al., 1996; Gunstone et al., 2007).

With fish oil supplementation, the *n*-3-PUFAs outstrip the arachidonic acids competitively in the enzyme-catalyzed eicosanoid synthesis pathway to generate eicosanoids of three-series prostaglandins, thromboxanes, and five-series leukotrienes, which are less proinflammatory (Wachtler et al., 1997; Calder and Grimble, 2002; Grimm et al., 2006). Therefore, reduced *n*-6:*n*-3 PUFA ratio of FOLE results in less-proinflammatory eicosanoid derivatives. Changes in the ratio of leukotrienes C_5_ to C_4_ production by peripheral blood mononuclear cells was highest when a mixture of 2:1 of the two FAs were used, and this ratio exerts the most favorable modulation of lipid mediator synthesis (Morlion et al., 1996).

## LEs in Formulation

The nature of oil used in LE formulations, depending on source and percentage contribution to the energy matrix, will determine the key functionalities between LEs. These differences account for their additional benefits or detrimental effects depending on prolonged use for critically ill or home PN patients. **Table 2** describes commercially available LEs and other components in PN for intravenous application. Soybean oil, predominantly *n*-6 PUFAs rich was combined with saturated MCTs identified as second generation LE. The third generation of LE primarily decreased the load of *n*-6 PUFAs by 75% with olive oil. By the year 2000, fish oil either alone or in combination with one or more of the based vegetable oils yielded the fourth generation LEs. Details of each LE will be discussed in the following sections. **Table 3** compares the concentrations of FAs from vegetable and fish oil sources. Their profile therefore varies in terms of therapeutic benefits and subsequent discussions reflect these aspects. 

Jadad scoring (Jadad et al., 1996) was applied to rate quality of cited randomized controlled trials evaluating various LEs formulations, and detailed in **Supplementary File** (**Table 1**). Overall, 8 studies (Gogos et al., 1990; Sedman et al., 1991; Ball, 1993; Jiang et al., 1993; Smirniotis et al., 1998; Mayer et al., 2003a; Sungurtekin et al., 2011; Demirer et al., 2016) were rated with a Jadad score of 1, 19 studies (Dionigi et al., 1985; Monson et al., 1986; Morlion et al., 1996; Battistella et al., 1997; Roulet et al., 1997; Waitzberg et al., 1997; Furukawa et al., 2002; Schauder et al., 2002; Weiss et al., 2002; Grau et al., 2003; Köller et al., 2003; Mayer et al., 2003b; Chen et al., 2005; Grimm et al., 2006; Iovinelli et al., 2007; Sabater et al., 2011; Ma et al., 2012; Zhu et al., 2012; Wu et al., 2014) scored 2, 11 studies (Wachtler et al., 1997; Chambrier et al., 1999; Linseisen et al., 2000; Garnacho-Montero et al., 2002; Wichmann et al., 2007; Berger et al., 2008; Barbosa et al., 2010; Onar et al., 2011; Wang et al., 2012; Gultekin et al., 2014; Chen et al., 2017) scored 3, 6 studies (Sandstrom et al., 1995; Kruimel et al., 2001; Lindgren et al., 2001; Vahedi et al., 2005; Wang et al., 2008; Piper et al., 2009) scored 4, and 11 studies (Bohnert et al., 2018; Klek et al., 2003; Senkal et al., 2007; Liang et al., 2008; Badia-Tahull et al., 2010; Jiang et al., 2010; Han et al., 2012; de Miranda Torrinhas et al., 2013; Metry et al., 2014; Grau-Carmona et al., 2015; Ma et al., 2015) scored 5.

**Table 2:** Commercially Available Lipid Emulsions in Parenteral Nutrition.

Type of LE	1st generation (1960s to 1970s) Soybean Oil LE	2nd generation (since 1985)		3rd generation (since 1990s) Olive Oil LE	4th generation (since 2000)		
		MCT/LCT physical mixture LE	Structured triglycerides LE		Pure fish Oil LE	MCT/SO/FO LE	SO/MCT/OO/FO LE
Oil source (% by wt)	100% SO	50% SO,50% CO	64% SO, 36% CO	20% SO, 80% OO	100% FO	50% CO, 40% SO, 10% FO	30% SO, 30% CO, 25% OO, 15% FO
Commercial name	Intralipid^®^ 20%	Lipofundin MCT/LCT^®^ 20%	Structolipid^®^ 20%	ClinOleic^®^ 20%	Omegaven^®^ 10%	Lipiderm/Lipoplus^®^ 20%	SMOFLipid^®^ 20%
Ratio of *n*-6:*n*-3 PUFAs	7:1^b,d^	7:1^b,d^	7:1^b,d^	9:1^b,d^	1:8^b,d^	2.7:1^d^	2.5:1^b,d^
Fat Content (g/L)	200^b^	200^b^	200^b^	200^c^	100^c^	200^a^	200^b^
Molecular weight	865^b^	634^b^	683^b^	873^b^	882^b^	NA^b^	732^b^
pH	8.0^b^	6.5–8.5^b^	8.0^c^	7.0–8.0^b^	7.5–8.7^b^	6.5–8.5*	8.0^b^
Osmolality(mOsmol/L)	350^b^	380^b^	350^b^	270^b^	273^b^	410*	380^b^
tocopherol (mg/L)	38^d^	85 ± 20^d^	6.9^d^	32^d^	150–296^d^	190 ± 30^d^	200^d^
Phytosterols (μcg/ml)	439.07 ± 5.72^e^	278.14 ± 5.09^e^	345.85 ± 1.64^e^	274.38 ± 2.6^e^	NR^e^	NR^e^	207^e^
FAC (% by weight of							
total FAs)							
SFA	15^b^	59.4^b^	46.3^b^	14.5^b^	21.2^b^	49–58.3^a,c^	36.9^c^
MUFA							
*OA*	24^b^	11^b^	14^b^	62.3^b^	15.1^b^	7.9–13.4^a,c^	30.8^c^
PUFA							
*LA*	44–62 ^b,d^	27–29.1^b,d^	35^b,d^	18.5–18.7^b,d^	4.4^b,d^	24.4–25.7^a,d^	21.4^d^
*α-LA*	4–11 ^b,d^	4–4.5^b,d^	5^b,d^	2–2.3^b,d^	1.8^b,d^	3.3–3.4^a,d^	2.5^d^
*AA*	0.1^b^	0.2^b^	NA^b^	0.5^b^	2.1^b^	0.5^c^	0.4^c^
*EPA*	NA^b,d^	NA^b,d^	NA^b,d^	NA^b,d^	19.2^b,d^	3.1–3.7^a,d^	3.0^d^
*DHA*	NA^b,d^	NA^b,d^	NA^b,d^	0.0–0.5^b,d^	12.1^,d^	2.3–2.5^a,d^	2.0^d^

AA, arachidonic Acid, CO, coconut oil; DHA, docosahexanoic acid; EPA, eicosapentaenoic acid, FAC, fatty acids concentration; FO, fish oil; LCT, long-chain triglycerides; LE, lipid emulsion; MCT, medium-chain triglycerides; MUFA, monounsaturated fatty acids; NA, not available; NR, not reported; OO, olive oil; PUFA, poly unsaturated fatty acids; SMOF, soybean oil, coconut oil, olive oil and fish oil; SFA, saturated fatty acids; SO, soybean oil.

^a^Linseisen et al., 2000.

^b^Wanten and Calder 2007.

^c^Driscoll et al., 2009.

^d^Vanek et al., 2012.

^e^Xu et al., 2012.

Fatty acid concentrations cited in the Table are reported as percentage by weight for the full product profiles but will not add up to 100% as only selected FAs are listed.

*Data provided is according to manufacturer monograph as per the lipid emulsion product.

**Table 3:** Fatty acid composition in selected plant and fish sources used in intravenous lipid emulsions.

FAC (% by weight)	Plant sources^*^			Fish species^#^					
	Soybean	Olive	Coconut	Atlantic mackerel^a^	Atlantic herring^b^	European anchovies^c^	Rainbow smelt^d^	Atlantic salmon^e^	Yellowfin tuna^f^
Caprylic acid (8:0)	NR/ND	NR/ND	8	NR/ND	NR/ND	NR/ND	NR/ND	NR/ND	NR/ND
Capric acid (10:0)	NR/ND	NR/ND	7	NR/ND	NR/ND	NR/ND	NR/ND	NR/ND	NR/ND
Lauric acid (12:0)	NR/ND	NR/ND	48	NR/ND	NR/ND	NR/ND	NR/ND	NR/ND	NR/ND
Myristic acid (14:0)	NR/ND	NR/ND	16	5.6	7.0	7.4	3.9	2.4	1.6
Palmitic acid (16:0)	11	10	9	17.6	17.1	17.4	16.6	11.2	23.2
Stearic acid (18:0)	4	2	2	NA	NA	NA	NA	NA	NA
Oleic acid (18:1)	23	78	7	18.9	19.2	15.2	20.6	24.0	16.1
Linoleic acid (18:2)	53	7	2	1.8	1.6	2.4	2.3	3.1	1.2
α-LA (18:3)	8	1	NR/ND	1.3	1.3	0	2.5	5.2	1.8
EPA (20:5)	NR/ND	NR/ND	NR/ND	7.4	8.9	13.1	13.9	5.7	5.4
DPA (22:5)	NR/ND	NR/ND	NR/ND	1.7	0.6	0.7	0.9	5.1	1.9
DHA (22:6)	NR/ND	NR/ND	NR/ND	11.6	10.8	22.2	21.1	19.8	26.8

α-LA, alpha-linolenic acid; DHA, docosahexanoic acid; DPA, docosapentaenoic acid; EPA, eicosapentaenoic acid; FAC, fatty acid concentration; NA; not available; ND; not detected; NR, not reported. FAC, fatty acids concentration based on percentage by weight, percentages do not add up to 100% because not all FAs are listed. Fish species from marine families of Carangidao, Clupeidae, Engraulidae, Osmeridae, Salmonidae, Scombridge (a–f).*Reference for plant sources: Iyer et al., 1998; Garnacho-Montero et al., 2002; Ma et al., 2012.^#^Reference for fish species: Driscoll et al., 2009.

### Soybean Oil-Based LEs

Soybean oil-based LEs (SOLEs) were the primary LEs in PN therapy, formulated with the objective of preventing the development of essential FA deficiency (Gramlich et al., 2015), as well as to offset the glucose load to prevent hyperglycemia (Schloerb, 2004). The total FA composition profile of soybean oil includes ~53% LA (18:2*n*-6), ~23% oleic acid, and ~8% α-linolenic acid (Gunstone et al., 2007). About 84% of soybean oil carries three 18-carbon, long-chain unsaturated FAs with the remaining ~15% from SFAs such as stearic acid ~4% and palmitic acid ~11% (Vanek et al., 2012; Kagawa et al., 2013). SOLEs have high *n*-6 PUFA compared to *n*-3 PUFA with ratio of 7:1 (Waitzberg et al., 2006). Commercially available LEs with 100% soybean oil is listed in **Table 2**.

Soybean oil is naturally rich in phytosterols, mainly ~60% β-sitosterol followed by ~20% each of campesterol and stigmasterol (Gunstone et al., 2007). Phytosterol concentration in soybean oil is ~320 mg/100 g oil (Verleyen et al., 2002). The abundance of phytosterols in SOLE is associated with the severity of* intestinal failure associated liver disease* in home PN patients (Ellegard et al., 2005; Llop et al., 2008). The concentration of α-tocopherol in soybean oil is ~7.5 mg/100 g oil but γ-tocopherol content ~80 mg/100 g oil (Gunstone et al., 2007) been greater, contributes to 10% of vitamin E’s biological activity (Biesalski, 2009).

#### Comparative Studies of SOLEs *vs* Lipid Free Glucose Infusions

**Table 4** reports on immuno-inflammatory and clinical outcomes of trials comparing the use of SOLE in PN with lipid free PN admixtures. Some studies reported either no change or immunostimulatory effects when SOLE was used compared to lipid-free PN admixtures in surgical patients (Dionigi et al., 1985; Monson et al., 1986; Li et al., 2007; Kagawa et al., 2013). Li *et al*. (2007) and Kagawa *et al*. (2012) suggested LE infusion is dose-dependent and the infusion rate at 0.09–0.12 g/kg/h does not alter human immune function (Li et al., 2007; Kagawa et al., 2013).

**Table 4:** Clinical outcomes of randomized controlled trials categorized as per lipid emulsion formulation type.

No.	Type of LE	Patient subgroups	Significant outcomes	References
1.	SOLE	Surgery	Nutrition	
			↑ glucagon, cumulative NB negative	(Furukawa et al., 2002)
			Immune and inflammatory	
			↑ PMN cells	(Dionigi et al., 1985)
			↑ IL-2	(Monson et al., 1986; Sedman et al., 1991)
			↓ LAK cell activity	(Sedman et al., 1991)
			↑ IL-6, ↑ CRP, ↓ lymphocyte proliferation	(Furukawa et al., 2002)
			↑ T cells and helper T cells, ↑ ADCC function	(Monson et al., 1986)
			↓ Bacteria killing mediated by neutrophils	(Waitzberg et al., 1997)
			Clinical	
			↓ Sepsis score	(Dionigi et al., 1985)
		ICU	Immune and inflammatory	
			↓ Helper/suppressor T cells ratio Clinical	(Gogos et al., 1990)
			↑ Infection, ↑ ICU stay, ↑ hospital stay, ↑ duration MV	(Battistella et al., 1997)
2.	Physical	Surgery	Nutrition	
	MCT/LCT		↓ Body weight, ↓ TG, ↑ β hydroxybutyrate	(Jiang et al., 1993)
			↑ Pre-albumin	(Chen et al., 2005)
			↑ Insulin	(Jiang et al., 1993; Chen et al., 2005)
			Immune and inflammatory	
			↑ NK and ↑ LAK cell activity Clinical	(Sedman et al., 1991)
			↓ Intra-abdominal infection	(Grau et al., 2003)
			↓ Mortality	(Grau et al., 2003)
		ICU	Nutrition	
			↓ Negative NB	(Ball, 1993; Garnacho-Montero et al., 2002)
			↑ Retinol binding protein, ↑ insulin	(Garnacho-Montero et al., 2002)
			Immune and inflammatory	
			No change in immune markers	(Iovinelli et al., 2007)
			Clinical	
			↑ Oxygen consumption, VO_2_	(Smirniotis et al., 1998)
			↓ Duration of MV	(Iovinelli et al., 2007)
3.	STG-LE	Surgery	Nutrition	
			No ↑ TG, No ↑ ASAT, No ↑ ALAT, NB positive	(Chambrier et al., 1999)
			Improved cumulative NB	(Chambrier et al., 1999; Kruimel et al., 2001)
			Less ↑ TG	(Kruimel et al., 2001)
			↑ Carbon dioxide production, ↑ whole-body fat oxidation, ↑ free fatty acid, ↑ plasma glycerol, ↑ 3-hydroxybutyric acid	(Sandstrom et al., 1995)
		ICU	Nutrition	
			Improved cumulative NB	(Lindgren et al., 2001)
4.	OOLE	HPN	Cellular fatty acid changes	
			↑ γ-LA, ↑ oleic acid, ↑ mead acid	(Vahedi et al., 2005)
		Surgery	Nutrition	
			↑ Body weight	(Demirer et al., 2016)
			↓ TBARS	(Demirer et al., 2016)
			↑ ALP, ↑ GGT, ↑ total protein, ↑ albumin, ↓ total bilirubin	(Onar et al., 2011)
			Clinical	
			No changes on catheter infections	(Onar et al., 2011)
5.	FOLE	Surgery	Nutrition	
			↑ α-Tocopherol	(Linseisen et al., 2000; Klek et al., 2003; Grimm et al., 2006; Wichmann et al., 2007)
			↓ AST, ↓ ALT	(Klek et al., 2003; Piper et al., 2009)
			↓ α-GST, ↓TG	(Piper et al., 2009)
			↓ LDL	(Ma et al., 2012)
			↑ APTT,	(Wang et al., 2012)
			↓ Total bilirubin,	(Klek et al., 2003; Wang et al., 2012)
			↓ Glucose	(Wu et al., 2014)
			Less ↓ HDL, less ↓ free fatty acid	(Ma et al., 2015)
			↑ Body weight, ↓ TBARS	(Demirer et al., 2016)
			Cellular fatty acid changes	
			↑ EPA or DHA	(Morlion et al., 1996; Roulet et al., 1997; Linseisen et al., 2000; Mayer et al., 2003b; Klek et al., 2003; Grimm et al., 2006; Senkal et al., 2007; Berger et al., 2008)
			↑ ALA	(Morlion et al., 1996)
			↑ EPA/AA ratio	(Roulet et al., 1997; Grimm et al., 2006; Senkal et al., 2007)
			↓ LA	(Linseisen et al., 2000)
			↑ Total *n*-3 PUFA, ↑ *n*-3:*n*-6 PUFA ratio	(Klek et al., 2003; Grimm et al., 2006; Senkal et al., 2007)
			Immune and inflammatory	
			↓ IL-6	(Clayton et al., 1993; Mayer et al., 2003b; Liang et al., 2008; Zhu et al., 2012; de Miranda Torrinhas et al., 2013)
			↓ LTB_4_	(Wachtler et al., 1997)
			↓ TNF-α	(Wachtler et al., 1997; Mayer et al., 2003b; Zhu et al., 2012)
			↑ TNF-α	(Schauder et al., 2002; Wang et al., 2012)
			↑ HLA-DR	(Clayton et al., 1993)
			↑ IL-2	(Schauder et al., 2002)
			↑ LTB_5_, ↑ LTB_5_/LTB_4_ ratio	(Wachtler et al., 1997; Köller et al., 2003; Grimm et al., 2006; Wichmann et al., 2007; Wang et al., 2012)
			↓ IL-1β, and ↓ IL-8	(Mayer et al., 2003b)
			↑ CD4+/CD8+	(Schauder et al., 2002; Liang et al., 2008)
			↓ CD4+/CD8+	(Zhu et al., 2012)
			↑ NF-Kβ	(Wang et al., 2012)
			↓ IL-10	(de Miranda Torrinhas et al., 2013)
			Clinical	
			↓ Post-operative stay on medical ward	(Clayton et al., 1993)
			↓ Length of hospital stay	(Grimm et al., 2006; Jiang et al., 2010; Zhu et al., 2012)
			↓ Infection rates	(Badia-Tahull et al., 2010)
			↓ Duration of SIRS	(Jiang et al., 2010; Zhu et al., 2012)
		ICU	Nutrition	
			↑ Albumin	(Gultekin et al., 2014)
			Cellular fatty acid changes	
			↑ EPA and DHA	(Mayer et al., 2003a; Mayer et al., 2003b; Wang et al., 2008; Barbosa et al., 2010)
			Immune and inflammatory	
			↓ IL-6	(Mayer et al., 2003b; Barbosa et al., 2010; Sungurtekin et al., 2011; Han et al., 2012; Metry et al., 2014)
			↓ TNF-α	(Mayer et al., 2003b; Barbosa et al., 2010; Sungurtekin et al., 2011; Han et al., 2012)
			↓ IL-10	(Barbosa et al., 2010; Sungurtekin et al., 2011)
			↑ Neutrophil inositol phosphate, ↑ PAF, ↑LTB_5_	(Mayer et al., 2003a)
			↑ Respiratory burst	(Mayer et al., 2003a)
			↓ IL-8	(Mayer et al., 2003b; Han et al., 2012)
			↓ CRP	(Wang et al., 2008; Gultekin et al., 2014)
			↓ IL-1β	(Mayer et al., 2003b; Barbosa et al., 2010)
			↓ IL-1	(Sungurtekin et al., 2011; Han et al., 2012)
			↓ LTB4	(Sabater et al., 2011; Gultekin et al., 2014)
			↓ IFN-γ	(Han et al., 2012)
			Clinical	
			Improvement in oxygenation index	(Wang et al., 2008; Barbosa et al., 2010)
			↓ CRRT days	(Wang et al., 2008)
			↓ Hospital stay	(Barbosa et al., 2010)
			↓ Nosocomial infections, ↑ predicted time free of infection	(Grau-Carmona et al., 2015)
			↓ 60-day mortality	(Chen et al., 2017)
		HPN	Cellular fatty acid changes	
			↑EPA,↑ DHA, ↑DPA in erythrocytes,platelets, serum phospholipids	(Bohnert et al., 2018)

AA, arachidonic acid; ADCC, antibody-dependent cellular cytotoxicity; ALA, α-linolenic acid; ALAT, alanine aminotransferase; ALP, alkaline phosphatase; ALT, alanine transaminase; APTT, activated partial thromboplastin time; ASAT/AST, aspartate aminotransferase; CD4/CD8+, T4 helper cells/T8 suppressor cells; CRP, C-reactive protein; CRRT, continuous renal replacement therapy; EPA, eicosapentaenoic acid; DHA, docosahexaenoic acid; DPA, docosapentaenoic acid; FOLE, fish oil lipid emulsion; GGT, gamma-glutamyl transferase; GST, glutathione S-transferase; HDL, high-density lipoprotein; HLA-DR, human leukocyte antigen-antigen D related; HPN, home parenteral nutrition; ICU, intensive care unit; IFN, interferon; Ig, immunoglobulin; IL, interleukin; LA, linoleic acid; LAK, lymphokine activated killer; LDL, low-density lipoprotein; LTB_4_, leukotriene B_4_; LTB_5_, leukotriene B_5_; MV, mechanical ventilation; NB, nitrogen balance; NK, natural killer; NF-Kβ, nuclear factor kappa β; OOLE, olive oil lipid emulsion, PAF, platelet activating factor; PMN, polymorphonuclear; PN, parenteral nutrition; PO_2_/FiO_2_, partial pressure of oxygen/fraction of inspired oxygen; PUFA, polyunsaturated fatty acid; SIRS, systemic inflammatory response syndrome; SOLE, soybean oil-based lipid emulsion; STG-LE, structured triglyceride lipid emulsion; TBARS, thiobarbituric acid-reactive substances; TG, triglyceride; TNF, tumor necrosis factor; VO_2_, oxygen consumption. For more details, please refer to **Supplementary File** (**Table 1**).

Evidence is conflicting associating SOLE use with clinical risk (**Table 4**) (Gogos et al., 1990; Sedman et al., 1991; Battistella et al., 1997; Waitzberg et al., 1997; Furukawa et al., 2002). Reductions in the ratio of helper to suppressor T-cells (Gogos et al., 1990), lymphokine-activated killer cell activity (Sedman et al., 1991), lymphocyte function (Battistella et al., 1997), lymphocyte proliferation (Furukawa et al., 2002), and bacteria killing by neutrophils (Waitzberg et al., 1997) have been reported. Post-surgical patients with severe stress, experienced increased interleukin-6 (IL-6) and C-reactive protein (CRP) levels after receiving SOLE compared to lipid-free PN. The high ratio of *n*–6:*n*–3 PUFAs in SOLE poses greater risk toward inflammatory response (Furukawa et al., 2002). LA metabolism promotes eicosanoid formation via enzymatic chain elongation, known to be proinflammatory mediators affecting macrophage, neutrophil and lymphocyte function (Wanten and Calder, 2007).

SOLE use is also associated with risk of adverse clinical outcomes. A meta-analysis reported higher infectious complication rates in surgical and intensive care patients on SOLE compared to lipid-free PN (Heyland et al., 1998). Polytrauma patients on SOLE for 10 days had increased number of days on mechanical ventilation, and longer stay in intensive care and hospital stay (Battistella et al., 1997). However one study reported reduced severity of infections in gastrointestinal surgery patients on SOLE compared to lipid-free PN (Dionigi et al., 1985). In contrast, patients undergoing bone marrow transplantation did not experience increased incidence of bacterial or fungal infections when infused with SOLE providing less than 30% energy/day (Lenssen et al., 1998).

##### Issues Associated With Use of SOLEs

The reticuloendothelial system participates in clearance of lipid particles associated with infusion of LEs. However, long-term administration of SOLE may deleteriously affect the reticuloendothelial system function (Seidner et al., 1989). Another risk of long-term use of SOLE is hepatotoxicity attributed to phytosterol content (Saubion et al., 1998; Llop et al., 2008).

### Physical Mixture MCT/LCT LEs

Concern about adverse effects from SOLE related to immuno-inflammatory and clinical risks led to the development of the physical mixture MCT/LCT. Physical mixtures combining MCT with LCT were initially formulated with coconut oil providing 50% of the MCT substrate with the remaining 50% from soybean oil. The MCT/LCT physical mixture formulation with effective manipulation of the percentage proportion of long-chain soybean oil with a shorter-chain FA, enabled the required reduction in the *n*-6 PUFA content in SOLEs. Coconut oil is the principal source of medium-chain FAs with lauric acid contributing 48% FA concentration, 8% caprylic acid, and 7% capric acid. Other fractions of FAs in coconut oil include 16% myristic acid, 9% palmitic acid, 2% stearic acid, 7% oleic acid and 2% LA (Gunstone et al., 2007). Typical commercial MCT/LCT LE formulations in use are detailed in **Table 2**.

Total phytosterol concentration in coconut oil (67.8 mg/100 g) is lower compared to soybean oil (~320 mg/100 g) with β-sitosterol contributing ~70%, followed by stigmasterol ~18% and campesterol ~11% (Verleyen et al., 2002). The α-tocopherol concentration of coconut oil is very low at 0.5 mg/100 g oil compared to soybean oil ~7.5 mg/100 g oil (Gunstone et al., 2007) which implies a lower phytosterol and α-tocopherol content in the physical mixture of MCT/LCT LEs, compared to SOLE.

#### Comparative Studies of MCT/LCT LEs With SOLEs

**Table 4** summarizes randomized controlled trials and crossover studies that have compared the effects of MCT/LCT LE and SOLE in abdominal surgery, critically ill, and long-term PN patients. Most of these studies concentrated on metabolic outcomes followed by clinical benefits. Two studies showed no influence of MCT/LCT LE on immune function in surgery and critically ill patients (Gogos et al., 1990; Sedman et al., 1991). In terms of immunomodulation there was no change in the ratio of helper to suppressor T-cells after MCT/LCT infusion, in contrast to a significant decrease in the ratio observed with SOLE (Gogos et al., 1990). In patients after gastrointestinal cancer surgery, MCT/LCT infusion increased natural killer cell activity and lymphokine activated killer activity compared to SOLE (Sedman et al., 1991). These findings are suggestive of improved immune function in the patient group receiving MCT/LCT LE. Indeed, MCTs do not depress the reticuloendothelial system function as evidenced from animal and human studies (Hamawy et al., 1985; Jensen et al., 1990). MCTs, being saturated cannot participate in eicosanoid synthesis and are not precursors for prostaglandins. Therefore they do not affect the production of inflammatory mediators during stress and illness in relation to *n*-6 PUFA (Radermacher et al., 1992).

Significant metabolic outcomes observed in intensive care and abdominal surgery patients administered with MCT/LCT LE infusion compared to SOLE are related to increase in plasma ketones (Ball and White, 1989; Jiang et al., 1993), non-esterified FAs (Ball and White, 1989), insulin (Jiang et al., 1993; Garnacho-Montero et al., 2002; Chen et al., 2005), prealbumin (Chen et al., 2005), retinol binding protein (Garnacho-Montero et al., 2002), and improved nitrogen balance (Ball, 1993; Garnacho-Montero et al., 2002). Contrarily one study did not observe any of these effects (Nijveldt et al., 1998).

Increased oxygen consumption in acute respiratory distress syndrome patients (Smirniotis et al., 1998), shorter weaning time from ventilator in chronic obstructive pulmonary disease patients (Iovinelli et al., 2007), and significant reduction in postoperative complications and mortality in preoperative surgery patients (Grau et al., 2003) are some clinical benefits attributed to MCT/LCT LE administration compared to SOLE.

##### Issues Associated With Use of MCT/LCT LEs

Since MCT oils are significantly low in essential FAs, dependency as a sole substrate of fat would be an issue in clinical applications unless combined with an *n*-6 PUFA-rich fat substrate to make up this deficit (Naber and Kruimel, 2002). A combination ratio of 1:1 for MCT and LCT is sufficient to prevent essential FA deficiency in long-term PN administration with an optimal dose of 0.41 ± 0.22 g/kg/day (Chambrier et al., 2004). Hypertriglyceridemia with initial infusion of MCTs is noted with MCT/LCT LE administration (Ball and White, 1989) and this is attributed to faster hydrolysis of MCTs. Additionally, generation of ketone metabolites, predominantly β-hydroxybutyrates (Ball, 1993) are associated with MCT/LCT LE and may induce increased insulin production (Naber and Kruimel, 2002).

### Structured Triglyceride LEs

The physical mixture of MCT/LCT LEs evolved further with the introduction of STGs as a new substrate option (Chambrier et al., 2006). The predominant commercial LE is a structured mix of 64% LCT from soybean oil and 36% of MCT from coconut oil (**Table 2**). STGs are used as a lipid source in 3-in-1 commercial PN Bag. 

Unlike physical mixtures, an STG-LE is formulated by first hydrolyzing soybean and coconut oils to free FAs, before interesterification in the presence of chemical or enzyme catalysts. Essentially medium-chain FAs (from coconut oil) and long-chain FAs (from soybean oil) are randomly reassigned on the glycerol backbone, generating new TAG molecular species (Karupaiah and Sundram, 2007). In STG-LEs, medium-chain FAs are preferentially positioned at the *sn*-1 and *sn*-3 positions of the TAG molecule with a long-chain FA occupying the *sn*-2 position (Chambrier et al., 2006). The choice of preferential placement of medium-chain FAs at the *sn-1* or *sn-3* position enables faster oxidation of energy substrates. An immunomodulating effect may be associated with STG if arachidonic acid, EPA, or DHA is incorporated at the *sn*-2 position (Chambrier et al., 2006). Additionally, favorable stereospecific positioning of medium-chain FAs in the molecular structure of STG facilitate faster triglyceride clearance and utilization as noted by Karupaiah and Sundram (2007). MCT/LCT LE carries more MCT compared to STG-LE (50% *vs* 36%) which fulfills the desired functionality of rapid hydrolysis compared to LCTs (Ball and White, 1989).

#### Comparative Studies of STG-LEs With SOLEs and Physical Mixture MCT/LCT LEs

**Table 4** reports on metabolic outcomes associated with STG-LE use compared to SOLE and MCT/LCT LE in surgery and critically ill patients. There is improved nitrogen retention in post-surgical patients receiving STG-LE compared to SOLEs and MCT/LCT LEs (Kruimel et al., 2001; Lindgren et al., 2001). Fat oxidation rate and 3-hydroxybutyric acid concentrations were found to be higher in the post-surgical STG-LE group, who received STG-LE for one day and SOLE the next day or *vice versa* for 6 days with lipid dose-dependence while maintaining amino acids and dextrose concentrations (Sandstrom et al., 1995). Conflicting findings related to differences in lipid infusion rates, switching of LE regimens daily, and different glucose:lipid ratios prescribed across study groups have been reported (Sandstrom et al., 1995; Bellantone et al., 1999; Chambrier et al., 1999).

The reduced risk of hypertriglyceridemia was noted in patients administered with STG-LE in post-surgical patients for 5 days (Chambrier et al., 1999; Kruimel et al., 2001) compared to MCT/LCT LEs. This effect could be attributed to favorable stereospecific positioning of FAs in the molecular structure of STG enabling faster triglyceride clearance and utilization (Karupaiah and Sundram, 2007).

Meta-analysis of 21 clinical trials with critically ill patients (n = 4) and surgical patients (n = 17) concluded short-term administration of STG-LE (5 to 7 days) was significantly associated with improved nitrogen balance, increased plasma proteins, reduced plasma triglycerides, improved liver function parameters, and reduced adverse events and length of hospital stay compared to physical mixture MCT/LCT LEs (Wu et al., 2017).

STG-LEs do not show any influence on the reticuloendothelial system, unlike the inhibition of reticuloendothelial system function shown by LCT (Chambrier et al., 2006).

##### Issues Associated With Use of STG-LEs

A lower α-tocopherol (6.9 mg/L) content adds to greater lipid peroxidation risk and higher phytosterols (~350 μg/ml) compared to MCT/LCT LE (~280 μg/ml), therefore adding to liver toxicity risk (Vanek et al., 2012; Xu et al., 2012). Naber and Krumel, (2002) observed STG-LE administration generated greater production of the ketone metabolite, β-hydroxybutyrate indicating a faster oxidation rate compared to physical mixture MCT/LCT (Naber and Kruimel, 2002). The impact of this metabolite on insulin and glycemic status of patients in therapy remains unreported.

### Olive Oil LEs

Another option to reduce *n*-6 PUFAs is with olive oil as a principal component by 80% replacement of soybean oil. The FA composition of olive oil reflects ~80% of the nonessential *n*–9 oleic acid with lesser proportions of the essential *n*–6 LA ~7% and the saturated palmitic acid ~10% (Gunstone et al., 2007). The *n*-3 PUFA in olive oil is negligible (**Table 2**). The small content of LA in olive oil explains the need to include an *n*-6 PUFA-rich source such as soybean oil to prevent essential FA deficiency. A formulation of olive oil LE (OOLE) blending ~20% soybean oil with ~80% olive oil in volume is commercially available (**Table 2**). Olthof *et al.* (2015) who studied 30 home PN patients receiving a combination of olive oil (80%) and soybean oil (20%) for 3 months at five times a week, reported their patients did not develop essential FA deficiency (Olthof et al., 2015).

Phytosterol concentration in olive oil is ~190 mg/100 g oil with 70% in the form of β-sitosterol (Verleyen et al., 2002). Olive oil also contains significant amounts of α-tocopherol (Sala-Vila et al., 2007) at ~12 mg/100 g oil (Gunstone et al., 2007), and thus provides sufficient protection against lipid peroxidation (Sala-Vila et al., 2007; Cai et al., 2018).

#### Comparative Studies of OOLEs With SOLEs and Physical Mixture MCT/LCT LEs

**Table 4** summarizes trials on OOLE compared to SOLE and physical mixture MCT/LCT LE in abdominal surgery, critically ill, and long-term PN patients. In terms of immuno-inflammatory outcomes, there was no difference in inflammatory, immune function markers, and pro-inflammatory cytokines (TNF-α and IL-6) in both critically ill (Umpierrez et al., 2012) and abdominal surgery (Demirer et al., 2016) patients randomized to receive either SOLE or MCT/LCT physical mixture *vs* OOLE. Similarly, inflammatory markers in home-PN patients on OOLE for 3 months were not different from baseline status (Reimund et al., 2005). Oleic acid predominant in olive oil, is hypothesized to offset immune system impairment when in combination with *n*-6 PUFA (Moussa et al., 2000; Cury-Boaventura et al., 2006). Metabolically, oleic acid does not influence the arachidonic eicosanoid pathway and is not a precursor for eicosanoids (Cai et al., 2018). Therefore a lesser proinflammatory response compared to soybean oil is likely.

Benefits on metabolic outcomes were reported by Onar et al. (2011). Significant increases in total protein and albumin followed by reduction in total bilirubin levels were reported in abdominal cancer surgery patients infused with OOLE for 7 days compared to SOLE. Weight gain has also been observed in patients receiving OOLE (Demirer et al., 2016). Home-PN patients with OOLE infusion over 2 months (Osowska et al., 2018), or 3 months (Reimund et al., 2005; Puiggros et al., 2009), post-abdominal surgery (Puiggros et al., 2009; Onar et al., 2011), and severely ill burns patients (Garcıa-de-Lorenzo et al., 2005) infused with OOLE did not have abnormal liver function tests. However evidence on clinical benefit of OOLE compared to MCT/LCT LE and STG-LE is still scarce.

The single unsaturated double-bond of oleic acid which characterizes OOLE likely mediates less lipid peroxidation and oxidative stress compared to LEs with *n*-6 and *n*-3 PUFAs (Sala-Vila et al., 2007). Animal studies (Dutot and Melin, 1991) and human studies with children (Goulet et al., 1999) and adults (Demirer et al., 2016) have reported reduction in thiobarbituric acid–reactive substances implying lowest risk of oxidative stress when given OOLE.

##### Issues Associated With Use of OOLEs

The impact of optimizing *n*-9 MUFA from OOLE is unknown in terms of whether there is competitive inhibition on the endogenous synthesis of *n*-3 PUFAs. This is because generation of gondoic acid and nervonic acid from oleic acid elongation have been reported to add on cardiovascular mortality risk (Delgado et al., 2017).

### Fish Oil LEs

Another option to reduce *n*-6 PUFA was to increase *n*-3 PUFAs with FOLE. Fish oil carries the characteristic longer chain *n*-3 PUFAs, EPA, and DHA in greater proportion compared to *n*–6 PUFAs. The FA concentration of fish oil comprises 1%–4% LA; 10%–30% of α-linolenic acid, EPA, and DHA; 5%–30% of oleic acid; and 18%–35% of SFAs (Gunstone et al., 2007). However, the nature of FA concentration depends on fish species used, as indicated in **Table 3** (Gunstone et al., 2007; Driscoll et al., 2009). Three different formulations of FOLEs have been commercialized as detailed in **Table 2**.

Phytosterol content is not an issue associated with fish oil use in LEs as fish oil is not plant based (Burrin et al., 2014). But the *n*-3 PUFAs been more prone to lipid peroxidation compared to *n*-6 PUFAs with more double bonds carried in their carbon chain (Linseisen et al., 2000). However the oxidative risk of FOLE is minimized by addition of α-tocopherol (Goulet et al., 2010). In fact, FOLEs compared to other LEs characteristically carry the highest α-tocopherol content ranging from 150 to 296 mg/L (**Table 2**).

FOLE administration after abdominal surgery is reported to modulate increased EPA content of plasma FA concentration (Morlion et al., 1996; Linseisen et al., 2000; Klek et al., 2003; Grimm et al., 2006; Senkal et al., 2007; Wichmann et al., 2007; Berger et al., 2008), platelets (Roulet et al., 1997; Bohnert et al., 2018), and erythrocytes (Klek et al., 2003; Senkal et al., 2007; Bohnert et al., 2018). Similarly, infusion of FOLE in sepsis patients reflected in increased *n*-3 FAs in plasma (Mayer et al., 2003a; Mayer et al., 2003b; Wang et al., 2008; Barbosa et al., 2010) and in mononuclear leukocyte membrane (Wachtler et al., 1997). This modulation of membrane lipids may favorably affect eicosanoids’ profile and other lipid mediators generated from arachidonic acid and EPA.

#### Comparative Studies of FOLE With SOLE, Physical Mixture MCT/LCT, and OOLE

**Table 4** summarizes trials related to FOLE for PN administration compared to SOLE, physical mixture MCT/LCT LE and OOLE in abdominal surgery and critically ill patients. Abdominal surgery patients receiving fish oil in the PN regimen demonstrated decreased production of inflammatory eicosanoids (Morlion et al., 1996; Wachtler et al., 1997; Köller et al., 2003; Grimm et al., 2006; Wichmann et al., 2007; Wang et al., 2012) and cytokines (Wachtler et al., 1997; Weiss et al., 2002; Liang et al., 2008; Zhu et al., 2012; de Miranda Torrinhas et al., 2013). This may offset the surgery-induced decline in antigen presenting cell activity (Weiss et al., 2002) and production of cytokines (Schauder et al., 2002). Modulation of inflammatory mechanisms and inflammatory mediator production appears to be attenuated in septic and acute respiratory distress syndrome patients (Mayer et al., 2003b; Barbosa et al., 2010; Sabater et al., 2011; Sungurtekin et al., 2011; Han et al., 2012; Gultekin et al., 2014; Metry et al., 2014). Pro-inflammatory cytokines TNF-α, IL-β, IL-6, and IL-8 secretion were shown to be significantly reduced by endotoxin-stimulated mononuclear cells in the FOLE group whereas an increase occurred 2 days after SOLE infusion (Mayer et al., 2003b). Significant reduced levels of inflammatory mediators IL-6 and TNF-α were noted in critically ill patients on FOLE compared to SOLE (161) or MCT/LCT LE (Barbosa et al., 2010; Sungurtekin et al., 2011; Han et al., 2012). Acute respiratory distress syndrome patients infused with FOLE in combination with MCT/LCT for 12 h had reduced leukotriene B4 production compared to SOLE alone (Sabater et al., 2011). Reduced leukotriene B4 production was also observed in septic patients receiving FOLE in combination with OOLE (160). In contrast, fish oil provided at varied doses before and after surgery in malignant large bowel surgical patients did not show any immunosuppressive benefit (Schauder et al., 2002). In patients undergoing abdominal aorta aneurysm repair (Berger et al., 2008) on MCT/LCT physical mixture or FOLE, no significant difference in the inflammatory marker CRP, in both groups of patients were noted.

Surgery patients administered with FOLE had higher plasma α-tocopherol concentration compared to SOLE, physical mixture MCT/LCT, or OOLE (Linseisen et al., 2000; Grimm et al., 2006; Wichmann et al., 2007). Other metabolic outcomes reported were significant improvement in liver enzymes (Klek et al., 2003; Piper et al., 2009), reduced triglycerides (Wu et al., 2014; Ma et al., 2015), total bilirubin (Wang et al., 2012), low-density lipoprotein-cholesterol (Ma et al., 2012), and weight gain (Demirer et al., 2016). However, only one study showed increased albumin levels in critically ill patients receiving FOLE infusion compared to OOLE (Gultekin et al., 2014).

Studies evaluating clinical benefit from FOLE administration compared to other LEs in surgical and critically ill patients report reduced mortality (Tsekos et al., 2004; Heller et al., 2006; Chen et al., 2017), shorter length of intensive care unit (ICU) (Heller et al., 2006) and hospital stay (Weiss et al., 2002; Tsekos et al., 2004; Grimm et al., 2006; Heller et al., 2006; Zhu et al., 2012), lesser mechanical ventilation (Tsekos et al., 2004), reduced infections (Badia-Tahull et al., 2010; Grau-Carmona et al., 2015), improved oxygenation index and reduction in renal replacement therapy days (Wang et al., 2008), reduced antibiotic demand (Heller et al., 2006), and decreased incidence and duration of systemic inflammatory response syndrome (Jiang et al., 2010; Zhu et al., 2012). Significant patient clinical outcomes were observed when fish oil was provided before surgery (Tsekos et al., 2004). However there are studies reporting no superior benefits for their critically ill and surgical patients (Mertes et al., 2006; Berger et al., 2008; Friesecke et al., 2008; Liang et al., 2008; Makay et al., 2011; Ma et al., 2012) when FOLE was prescribed with PN admixtures. Improvement in clinical outcomes is likely associated with dose of fish oil in PN (Tsekos et al., 2004; Heller et al., 2006). Heller et al. (2006) observed reduced mortality with FOLE dosed at 0.1 g/kg/day, shorter length of ICU and hospital stays with FOLE doses between 0.1 and 0.2 g/kg/day, and antibiotic demand reduced by 23% with infusion doses of 0.15–0.2 g/kg/day (Heller et al., 2006).

##### Issues Associated With Use of FOLEs

Since pure FOLE carries *n*-6 PUFA minimally, there is potential risk for essentially FA deficiency in patients administered with this emulsion as monotherapy. However some studies demonstrate patient success with single-therapy pure FOLE in pediatrics (Gura et al., 2005; De Meijer et al., 2009; Le et al., 2011). It is suggested that essential FA deficiency prevention is possible with pure FOLE dosed at 1 g/kg/day (Gura et al., 2005; Le et al., 2011).

## Conclusions

This review relates fundamental knowledge on FA composition, functionality, and clinical evidence of lipids used in PN, which should be appreciated for future LE formulations. Clearly, LEs have evolved from just been a primary source of energy for PN to becoming therapeutic substrates capable of targeted outcomes in critically ill patients. It appears FA composition of LEs is the primary determinant of desired clinical outcomes, matched to its biological function. Options for LE choice becomes more specific based on performance outcomes as indicated for immuno-inflammatory, metabolic and clinical outcomes. Additionally the final composition of LE formulations must take into consideration the potential for essential FA deficiency, lipid peroxidation, phytosterol toxicity, hypertriglyceridemia, and hyperglycemia. It appears therefore, one type of LE alone cannot be uniformly applied to patients undergoing medical interventions.

We note that at this point, most clinical trials evaluating LEs describe only limited clinical outcomes. More studies reported the physical mixture MCT/LCT, STG-LE, and FOLE administrations benefit metabolic outcomes whereas studies on SOLE indicate greater risk on immuno-inflammatory outcomes. Current evidence is insufficient to support clinical benefits associated with FOLE use compared to OOLEs but some studies do report that fish oil included in PN may be beneficial for critically ill patients. More studies adequately powered, with standardized dosage and longer duration of supplementation are needed to indicate clear benefit of FOLE in hyperinflammatory states.

## Author Contributions

BS, SN, and TK were involved in all processes of the review from literature search, study selection, and data extraction. BS and BK performed quality assessment. BS, SN, and BK finalized tables. BS, SN, SS, BK, and TK drafted the manuscript. AA, EF, and KS contributed to the writing and critical revision of the manuscript. All authors read, revised, and approved the final manuscript.

## Funding

This work is fully supported by the Fundamental Research Grant Scheme (FRGS/1/2016/SKK03/UKM/01/1) from the Ministry of Higher Education, (MOHE) Malaysia. BS is a recipient of a PhD study scholarship from Universiti Kebangsaan Malaysia and BK is a PhD Zamalah scholar under the Vice Chancellor of UKM. SS is a recipient of mybrain scholarship from MOHE.

## Conflict of Interest

Co-author KS is employed at the Malaysian Palm Oil Council. He has published widely in lipid science.

The remaining authors declare that the research was conducted in the absence of any commercial or financial relationships that could be construed as a potential conflict of interest.
