# Corrigendum: Composition and Functionality of Lipid Emulsions in Parenteral Nutrition: Examining Evidence in Clinical Applications

**DOI:** 10.3389/fphar.2021.663960

**Published:** 2021-04-16

**Authors:** Birinder Kaur Sadu Singh, Sreelakshmi Sankara Narayanan, Ban Hock Khor, Sharmela Sahathevan, Abdul Halim Abdul Gafor, Enrico Fiaccadori, Kalyana Sundram, Tilakavati Karupaiah

**Affiliations:** ^1^ Nutrition Programme, Faculty of Health Sciences, National University of Malaysia, Kuala Lumpur, Malaysia; ^2^ Faculty of Health and Medical Science, School of BioSciences, Taylor’s University Lakeside Campus, Selangor, Malaysia; ^3^ Dietetics Programme, Faculty of Health Sciences, National University of Malaysia, Kuala Lumpur, Malaysia; ^4^ Medical Department, Faculty of Medicine, Universiti Kebangsaan Malaysia Medical Centre, Kuala Lumpur, Malaysia; ^5^ Acute and Chronic Renal Failure Unit, Department of Clinical and Experimental Medicine, University of Parma, Parma, Italy; ^6^ Malaysian Palm Oil Council (MPOC), Petaling Jaya, Malaysia

**Keywords:** fatty acids, lipid emulsions, triglycerides, triacylglycerols, parenteral nutrition

In the original article, there was a mistake in the legend for *Table 2* as published. Referring to the references cited in the original article Table 2, Linseisen et al., 2000 and Driscoll et al., 2009 were missing, while Waitzberg et al., 2006 has to be removed. Therefore, Linseisen et al., 2000 and Driscoll et al., 2009 were added as the reference for the corrected Table 2. We added a sentence for the footnote of corrected Table 2: “Fatty acids concentration cited in the Table are reported as percentage by weight for the full product profiles but will not add up to 100% as only selected FAs are listed. In addition, a sentence “Data provided is according to manufacturer monograph as per the lipid emulsion product” was added as the footnote for the corrected Table 2. The correct legend appears below. The authors apologize for this error and state that this does not change the scientific conclusions of the article in any way. The original article has been updated.

**TABLE 2:** Commercially Available Lipid Emulsions in Parenteral Nutrition.

Type of LE	1st generation (1960s–1970s) Soybean oil LE	2nd generation (since 1985)		3rd generation (since 1990s) Olive oil LE	4th generation (since 2000)		
		MCT/LCT physical mixture LE	Structured triglycerides LE		Pure fish oil LE	MCT/SO/FO LE	SO/MCT/OO/FO LE
Oil source (% by wt)	100% SO	50% SO, 50% CO	64% SO, 36% CO	20% SO, 80% OO	100% FO	50% CO, 40% SO, 10% FO	30% SO, 30% CO, 25% OO, 15% FO
Commercial name	Intralipid® 20%	Lipofundin MCT/LCT® 20%	Structolipid® 20%	ClinOleic® 20%	Omegaven® 10%	Lipiderm/Lipoplus® 20%	SMOFLipid® 20%
Ratio of *n*-6:*n*-3 PUFAs	7:1^b^ ^,^ ^d^	7:1^b^ ^,^ ^d^	7:1^b^ ^,^ ^d^	9:1^b^ ^,^ ^d^	1:8^b^ ^,^ ^d^	2.7:1^d^	2.5:1^b^ ^,^ ^d^
Fat content (g/L)	200^b^	200^b^	200^b^	200^c^	100^c^	200^a^	200^b^
Molecular weight	865^b^	634^b^	683^b^	873^b^	882^b^	NA^b^	732^b^
pH	8.0^b^	6.5–8.5^b^	8.0^c^	7.0–8.0^b^	7.5–8.7^b^	6.5–8.5^*^	8.0^b^
Osmolality (mOsmol/L)	350^b^	380^b^	350^b^	270^b^	273^b^	410^*^	380^b^
tocopherol (mg/L)	38^d^	85 ± 20^d^	6.9^d^	32^d^	150–296^d^	190 ± 30^d^	200^d^
Phytosterols (μcg/ml)	439.07 ± 5.72^e^	278.14 ± 5.09^e^	345.85 ± 1.64^e^	274.38 ± 2.6^e^	NR^e^	NR^e^	207^e^
FAC (% by weight of total FAs)						
SFA	15^b^	59.4^b^	46.3^b^	14.5^b^	21.2^b^	49–58.3^a^^,^ ^c^	36.9^c^
MUFA						
*OA*	24^b^	11^b^	14^b^	62.3^b^	15.1^b^	7.9–13.4^a^^,^ ^c^	30.8^c^
PUFA						
*LA*	44–62^b^ ^,^ ^d^	27–29.1^b^ ^,^ ^d^	35^b^ ^,^ ^d^	18.5–18.7^b^ ^,^ ^d^	4.4^b^ ^,^ ^d^	24.4–25.7^a^^,^ ^d^	21.4^d^
α-*LA*	4–11^b^ ^,^ ^d^	4–4.5^b^ ^,^ ^d^	5^b^ ^,^ ^d^	2–2.3^b^ ^,^ ^d^	1.8^b^ ^,^ ^d^	3.3–3.4^a^^,^ ^d^	2.5^d^
*AA*	0.1^b^	0.2^b^	NA^b^	0.5^b^	2.1^b^	0.5^c^	0.4^c^
*EPA*	NA^b^ ^,^ ^d^	NA^b^ ^,^ ^d^	NA^b^ ^,^ ^d^	NA^b^ ^,^ ^d^	19.2^b^ ^,^ ^d^	3.1–3.7^a^^,^ ^d^	3.0^d^
*DHA*	NA^b^ ^,^ ^d^	NA^b^ ^,^ ^d^	NA^b^ ^,^ ^d^	0.0–0.5^b^ ^,^ ^d^	12.1^d^	2.3–2.5^a^^,^ ^d^	2.0^d^

AA, arachidonic Acid; CO, coconut oil; DHA, docosahexanoic acid; EPA, eicosapentaenoic acid; FAC, fatty acids concentration; FO, fish oil; LCT, long-chain triglycerides; LE, lipid emulsion; MCT, medium-chain triglycerides; MUFA, monounsaturated fatty acids; NA, not available; NR, not reported; OO, olive oil; PUFA, polyunsaturated fatty acids; SMOF, soybean oil, coconut oil, olive oil and fish oil; SFA, saturated fatty acids; SO, soybean oil.

^a^ Linseisen et al., 2000.

^b^ Wanten and Calder 2007.

^c^ Driscoll et al., 2009.

^d^ Vanek et al., 2012.

^e^ Xu et al., 2012.

Fatty acid concentrations cited in the Table are reported as percentage by weight for the full product profiles but will not add up to 100% as only selected FAs are listed.

^*^ Data provided is according to manufacturer monograph as per the lipid emulsion product.

**References:** “^a^Linseisen et al., 2000; ^b^Wanten and Calder 2007; ^c^Driscoll et al., 2009; ^d^Vanek et al., 2012; ^e^Xu et al., 2012.”

In the original article, there was a mistake in *Table 2* as published. The Lipiderm/Lipoplus^®^ detailed fatty acids composition was referred from Linseisen et al., 2000 and this reference was missed out in the original Table 2. The authors cross-checked and recalculated the data on fatty acid composition presented in Table 2 based on one particular cited paper (Vanek et al., 2012), which indicated there are errors in the extrapolation of the data from the primary source (Waitzberg et al., 2006). Therefore the authors acknowledged these errors and amended the data accordingly to reflect percent composition of fatty acids by full profile (Driscoll et al., 2009). The corrected *Table 2* appears below. The authors apologize for this error and state that this does not change the scientific conclusions of the article in any way. The original article has been updated.

