# Supplementary material for: Composition and Functionality of Lipid Emulsions in Parenteral Nutrition: Examining Evidence in Clinical Applications
Source: Front Pharmacol. 2020 Apr 29;11:506. doi: 10.3389/fphar.2020.00506 (PMC7201073; doi:10.3389/fphar.2020.00506)
Supplement: Supplementary file 1 [file Table_1.pdf]

Supplementary Material

TABLE S1 Clinical Outcomes of Randomized Controlled Trials categorized as per Lipid Emulsion Formulation Type

No	Cited Studies	Patient Type	*n* (IG/CG)	Control Group	Duration (days)	IIO*	NO*	CO*	Cellular FAC Changes*	Significant Outcomes*	Jadad Score†
					**RCTs with Soybean Oil Lipid Emulsion (SOLE)**						
1	Dionigi *et al*, 1985	Malnourished GI cancer surgery	8/7	Lipid Free PN	21	+, -	NR	+	NR	↑ PMN cells for IG group.<br><br>No change in IgG, IgM, C3c, Factor B for IG.<br><br>↓ sepsis score for IG.	**2**
2	Gogos *et al*, 1990	Malnourished critically ill	20/20	Lipid Free PN	10	-	NR	NR	NR	↓ ratio of helper to suppressor T cells for IG	**1**
3	Sedman *et al*, 1991	Malnourished GI cancer surgery	12/9	Lipid Free PN	7	+, -	NR	NR	NR	↓ LAK cell activity, ↑ IL-2 activity for IG.	**1**
4	Battisela *et al,* 1997	Polytrauma	30/27	Lipid Free PN	10	NR	NR	-	NR	↑ infections, ↑ ICU stay, ↑ hospital stay,<br><br>↑ duration MV for IG.	**2**
5	Furukawa *et al*, 2002	Gastric, Colorectal esophageal surgery	1717	Lipid Free PN	21	-	-	NR	NR	↑ IL-6 & ↑ CRP, ↓ lymphocyte proliferation in severe stress IG.<br><br>↑ Glucagon, cumulative NB negative in severe stress IG.	**2**
6	Monson *et al,* 1986	Malnourished, septic, cancer surgery	12/11	Lipid Free PN	14	+	NR	NR	NR	↑ IL2, ↑ T cells, & Helper T cells, ↑ ADCC function for IG.	**2**
7	Waitzberg *et al,* 1997	Malnourished GI cancer surgery	10/10	Lipid Free PN	2	-	NR	NR	NR	↓ bacteria killing mediated by neutrophils for IG.	**2**

TABLE S1 Continued

No	Cited Studies	Patient Type	*n* (IG/CG)	Control Group	Duration (days)	IIO*	NO*	CO*	Cellular FAC Changes*	Significant Outcomes*	Jadad Score
					**RCTs with Physical Mixture MCT/LCT Lipid Emulsion**						
8	Sedman *et al*, 1991	Malnourished GI cancer surgery	12/12	SOLE	7	+	NR	NR	NR	↑ NK & ↑ LAK cell activity for IG.	**1**
9	Ball 1993	Ventilated ICU	10/10	SOLE	8	NR	+	NR	NR	↓ negative NB for IG	**1**
10	Jiang *et al*, 1993	Abdominal surgery	6/6	SOLE	10	NR	+, -	NR	NR	↓ Body Weight, ↑ insulin, ↓ TG, ↑ β hydroxybutyrate for IG	**1**
11	Smirniotis *et al*, 1998	Post operative, ICU, sepsis, ARDS	11/10	SOLE	1	NR	NR	+	NR	↑ oxygen consumption, VO_2 for IG.	**1**
12	Garnacho-Montero *et a*l, 2002	ICU with sepsis	26/26	SOLE	10	NR	+	NR	NR	↑ Retinol Binding Protein, ↑ insulin, improved NB for IG	**3**
13	Grau *et a*l, 2003	Malnourished surgery & cancer	26/31	SOLE	5	NR	NR	+	NR	↓ intra-abdominal infection for IG.<br>↓ mortality in IG group without cancer	**2**
14	Chen *et al*, 2005	GI surgery	15/15	SOLE	7	NR	+	NR	NR	↑ pre-albumin, ↑ insulin for IG	**2**
15	Iovinelli *et al*, 2007	COPD on MV in ICU	7/7	SOLE	15	+,-	NR	+	NR	No change in immune markers for IG<br>↓ time of MV for IG	**2**
					**RCTs with Structured Triglycerides Lipid Emulsion (STGLE)**						
16	Chambrier *et a*l, 1999	Post abdominal surgery	19/19	MCT/ LCT	5	NR	+	NR	NR	No ↑ TG, No ↑ ASAT, No ↑ ALAT, NB positive for IG	**3**
17	Kruimel *et al*, 2001	Postoperative aortic prosthesis	12/13	MCT/ LCT	5	NR	+	NR	NR	Improved cumulative NB, Less ↑ TG for IG	**4**

TABLE S1 Continued

No	Cited Studies	Patient Type	*n* (IG/CG)	Control Group	Duration (days)	IIO*	NO*	CO*	Cellular FAC Changes*	Significant Outcomes*	Jadad Score†
18	Lindgren *et al*, 2001	ICU	9/11	SOLE	5	NR	+	NR	NR	Improved cumulative NB for IG	**4**
19	Sandstrom *et al*, 1995	Post abdominal surgery	19/18	SOLE	6	NR	+,-	NR	NR	↑ carbon dioxide production, ↑ whole-body fat oxidation, ↑ Free Fatty Acid, ↑ plasma glycerol, ↑ 3-hydroxybutyric acid, with STGLE in part 2	**4**
					**RCTs with Olive Oil Lipid Emulsion (OOLE)**						
20	Vahedi *et al*, 2005	Home parenteral nutrition with intestinal failure	7/6	SOLE	90	NR	NR	NR	+	↑ Ɣ-LA, ↑ oleic acid, ↑ mead acid in plasma for IG	**4**
21	Onar *et al*, 2011	Abdominal oncologic surgery	10/10	SOLE	7	NR	+,-	+,-	NR	↑ ALP, ↑ GGT, ↑ total protein, ↑ albumin, ↓ total bilirubin for IG<br>No changes on catheter infections for IG	**3**
22	Demirer *et al*, 2016	Elective GI surgery	13/18	MCT/LCT	4	NR	NR	+	NR	↑ Weight, ↓ TBARS for IG	**1**
					**RCTs with Fish Oil Lipid Emulsion (FOLE)**						
23	Morlion 1996	Abdominal surgery	10/10	SOLE	5	NR	NR	NR	+	↑ plasma phospholipid EPA, DHA and ALA for IG	**2**
24	Wachtler *et al*, 1997	Major intestinal surgery	20/20	MCT/LCT	5	+	NR	NR	NR	↑ LTB_5, ↓ LTB_4, ↓ LTB_4/LTB_5 ratio, ↓ TNF-α for IG	**3**
25	Roulet *et al*, 1997	Elective total esophagectomy	10/9	SOLE	7	NR	NR	NR	+	↑ EPA and EPA/AA in platelet phospholipids for IG	**2**
26	Linseisen *et al,* 2000	Major abdominal cancer surgery	17/16	SOLE	5	NR	+	NR	+	↑ α-tocopherol, ↓ Ɣ- tocopherol for IG<br>↑ plasma EPA, ↓ LA for IG	**3**

TABLE S1 Continued

No	Cited Studies	Patient Type	*n* (IG/CG)	Control Group	Duration (days)	IIO*	NO*	CO*	Cellular FAC Changes*	Significant Outcomes*	Jadad Score†
27	Weiss *et a*l, 2002	Elective abdominal surgery	12/11	SOLE	6	+	NR	+	NR	↓ IL-6, ↑ HLA-DR for IG<br>↓ post-operative stay on medical ward for IG	**2**
28	Schauder *et al*, 2002	Large bowel surgery	19/20	SOLE	6	+,-	NR	NR	NR	↑ IL-2, ↑ TNF, ↑ CD4/CD8 + for IG	**2**
29	Koller *et al*, 2003	Elective abdominal surgery	14/16	SOLE	5	+	NR	NR	NR	↑ in LTB_5, ↑ LTB_5/LTB_4 ratio for IG	**2**
30	Grimm *et al*, 2006	Major abdominal surgery	19/14	SOLE	5	+	+	+	+	↑ in LTB_5, ↑ LTB_5/LTB_4 ratio, ↑ α-tocopherol, ↓ length of hospital stay, ↑ total *n*-3 FA, ↑ plasma EPA & DHA, ↑ EPA/AA ratio, ↑ *n*-3:*n*-6 ratio for IG	**2**
31	Senkal *et al*, 2007	Elective colorectal surgery	19/21	MCT/LCT	5	NR	NR	NR	+	↑ EPA & DHA, ↑ *n*-3:*n*-6 ratio for IG	**5**
32	Wichmann *et al*, 2007	Major abdominal surgery	127/ 129	SOLE	5	+	+	NR	NR	↑ in LTB_5, ↑ LTB_5/LTB_4 ratio for IG<br>↑ α-tocopherol for IG	**3**
33	Liang *et al*, 2008	Radical colorectal cancer surgery	20/21	SOLE	7	+	NR	NR	NR	↓ IL-6, ↑ CD4+/CD8+ for IG	**5**
34	Berger *et al*, 2008	Abdominal aortic aneurysm	12/12	MCT/LCT	4	NR	NR	NR	+	↑ in EPA & DHA for IG	**3**
35	Piper *et al*, 2009	Post abdominal surgery or cancer	22/22	OOLE	5	NR	+	NR	NR	↓ AST, ↓ ALT, ↓ α-GST, ↓ TG for IG	4

TABLE S1 Continued

No	Cited Studies	Patient Type	*n* (IG/CG)	Control Group	Duration (days)	IIO*	NO*	CO*	Cellular FAC Changes*	Significant Outcomes*	Jadad Score†
36	Badia-Tahull *et al*, 2010	Elective GI surgery	13/14	OOLE	5	NR	NR	+	NR	↓ Infection rates for IG	**5**
37	Jiang *et al*, 2010	GI/colonic cancer	100/ 103	SOLE	7	NR	NR	+	NR	↓ SIRS, ↓ hospital stay for IG	**5**
38	Ma *et al*, 2012	GI tumour surgery	20/20	MCT/LCT	5	NR	+	NR	NR	↓ Low Density Lipoprotein for IG	**2**
39	Zhu *et a*l, 2012	Colorectal cancer surgery	29/28	SOLE	7	+,-	NR	+	NR	↓ CD4+/CD8+, ↓ IL-6, ↓ TNF- α for IG<br>↓ duration of SIRS, ↓hospital stay	**2**
40	Wang *et al*, 2012	GI surgery	32/32	MCT/LCT	5	+,-	+,-	NR	NR	↑ TNF-α, ↑ NF-K β , ↑ LTB_5/LTB_4 ratio for IG<br>↑ APTT, ↓ total bilirubin for IG	**3**
41	Klek *et al*, 2013	Intestinal failure	34/39	SOLE	28	NR	+	NR	+	↓ AST, ↓ ALT ↓ total bilirubin, ↑ α-tocopherol for IG<br>↑ EPA & DHA in plasma & RBC, ↓ *n*-6:*n*-3 ratio for IG	**5**
42	De Miranda Torrinhas *et al*, 2013	GI cancer	31/32	MCT/LCT	3	+	NR	NR	NR	↓ IL-6, ↓ IL-10 for IG	**5**
43	Wu *et al*, 2014	GI surgery	20/15	MCT/LCT	5	NR	+	NR	NR	Less ↑ TG & ↓ Glucose for IG	**2**
44	Ma *et al*, 2015	Gastric, Colorectal cancer surgery	45/41	MCT/LCT	8	NR	+	NR	NR	Less ↑ TG, Less ↓HDL, Less ↓ Free Fatty Acid for IG	**5**
45	Demirer *et al*, 2016	Elective GI surgery	21/18	MCT/LCT	4	NR	+	NR	NR	↑ body weight, ↓ TBARS for IG	**1**

TABLE S1 Continued

No	Cited Studies	Patient Type	*n* (IG/CG)	Control Group	Duration (days)	IIO*	NO*	CO*	Cellular FAC Changes*	Significant Outcomes*	Jadad Score*
46	Mayer *et al*, 2003	Sepsis	5/5	SOLE	10	+	NR	NR	+	↑ Neutrophil Inositol Phosphate, ↑ PAF, ↑ LTB_5, for IG<br>↑ respiratory burst for IG,<br>↑ EPA & DHA in plasma for IG	**1**
47	Mayer *et a*l, 2003	Sepsis	10/9	SOLE	5	+	NR	NR	+	↓ TNF-α, ↓ IL-1B, ↓ IL-6 and ↓ IL-8 for IG<br>↑ EPA & DHA in plasma & leukocytes for IG	**2**
48	Wang *et al*, 2008	Acute pancreatitis	20/20	SOLE	5	+	NR	+	+	↓ CRP, improvement in oxygenation index for IG<br>↓ CRRT days, ↑ plasma EPA for IG	**4**
49	Barbosa *et al*, 2010	SIRS/Sepsis	13/10	MCT/LCT	6	+	NR	+	+	↓ IL-6, ↓ IL-10, ↓ TNF-α, ↓ IL-1 β for IG<br>↓ hospital stay, ↑ PO2/FiO2, ↑ EPA in plasma for IG	**3**
50	Sungurtekin *et al*, 2011	SIRS/Sepsis	10/10	MCT/LCT	7	+,-	NR	NR	NR	↑ IL-10, ↓ IL-6, ↓ TNF-α, ↓ IL-1 for IG with sepsis	**1**
51	Sabater *et al*, 2011	ARDS	8/8	SOLE	12 hours	+	NR	NR	NR	↓ LTB4 for IG	**2**
52	Han *et al*, 2012	Surgical ICU	18/12	MCT/LCT	7	+	NR	NR	NR	↓ IL-1, ↓ IL-8, ↓ IFN-ɣ, ↓ IL-6, ↓ TNF-α for IG	**5**
53	Gultekin *et al*, 2014	Sepsis	16/16	OOLE	5	+	+	NR	NR	↓ LTB4, ↓ CRP, ↑ albumin for IG	**3**
54	Metry *et al*, 2014	Post-surgery ICU	41/42	SOLE	7	+	NR	NR	NR	↓ IL-6 for IG	**5**
55	Grau-Carmona *et al*, 2015	ICU	81/78	MCT/LCT	5	NR	NR	+	NR	↓ nosocomial infections, ↑ predicted time free of infection for IG	**5**

TABLE S1 Continued

No	Cited Studies	Patient Type	*n* (IG/CG)	Control Group	Duration (days)	IIO*	NO*	CO*	Cellular FAC Changes*	Significant Outcomes*	Jadad Score†
56	Chen *et al*, 2017	Sepsis	41/37	SOLE	7	NR	NR	+	NR	↓ 60 day mortality for IG	**3**
57	Bohnert *et al*, 2018	Home Parenteral Nutrition	15/18	MCT/LCT	56	-	-	-	+	↑ EPA, ↑ DHA, ↑ DPA in erythrocytes, platelets, serum phospholipids for IG	**5**

AA, Arachidonic Acid; ADCC, Antibody-dependent cellular cytotoxicity; ALA, Alpha Linolenic Acid; ALAT, Alanine aminotransferase; ALP, Alkaline phosphatase; ALT, Alanine transaminase; APTT, Activated Partial Thromboplastin Time; ASAT/AST, Aspartate aminotransferase; CD4/CD8+, T4 helper cells/T8 suppressor cells; CG, Control Group; CO, Clinical Outcome; CRP, C-Reactive Protein; CRRT, Continuous Renal Replacement Therapy; DHA, docosahexaenoic acid; DPA, Docosapentaenoic acid; EPA, Eicosapentaenoic acid; FAC, Fatty Acid Composition; GGT, Gamma-Glutamyl Transferase; GI, Gastrointestinal; GST, Glutathione S-transferase; HLA-DR, Human Leukocyte Antigen-antigen D related;ICU, intensive care unit; IFN, Interferon; Ig, Immunoglobulin; IG, Intervention Group; IIO, Immuno-Inflammatory Outcome; IL, Interleukin; LA, Linoleic Acid; LAK, Lymphokine Activated Killer; LTB_4, Leukotriene B_4; LTB_5, Leukotriene B_5; MV, Mechanical Ventilation; NB, Nitrogen Balance; NF-Kβ,nuclear factor-kB; NK, Natural Killer; NO, Nutrition Outcome; PAF, Platelet Activating Factor; PMN, Polymorphonuclear; PN, Parenteral Nutrition; PO2/FiO2, partial pressure of oxygen/fraction of inspired oxygen; SIRS, Systemic Inflammatory Response Syndrome; TBARS, Thiobarbituric Acid Reactive Substances; TG, Triglyceride; TNF, Tumour Necrosis Factor.

Footnote:

*"+",benefit; "-",risk'; NR, Not Reported; "↓", significantly reduced; "↑", significantly increased.

†Jadad Score an instrument used to limit the risk of introducing bias and assess quality of RCTs. Scoring was given based on RCTs described as randomized, double-blinded and withdrawals or drop-outs were stated. Additional points were given if method to generate the sequence of randomization and double-blinding was described and it was appropriate. However points will be deducted if the method to generate the sequence of randomization and double-blinding was described but was inappropriate. Additionally if withdrawals were not mentioned, no points will be given. High quality RCTs are scored at 4- 5 and low quality at 1-2.
